# Missed Autoimmune Diabetes: Latent Autoimmune Diabetes in Adults in the Setting of Autoimmune Clustering

**DOI:** 10.7759/cureus.102728

**Published:** 2026-01-31

**Authors:** Saraswathi Saiprasad, Narayana Swamy

**Affiliations:** 1 Endocrinology, Diabetes and Metabolism, Baylor Scott & White Health, Fort Worth, USA; 2 Rheumatology, Baylor Scott & White Health, Fort Worth, USA

**Keywords:** autoimmune clustering, autoimmune diabetes, diabetic cheiroarthropathy, hashimoto thyroiditis, insulin therapy, lada, latent autoimmune diabetes in adults, misdiagnosis, pancreatic autoantibodies

## Abstract

Latent autoimmune diabetes in adults (LADA) is frequently misdiagnosed as type 2 diabetes mellitus (T2DM), particularly in older adults, owing to its adult onset, initial insulin independence, and indolent clinical course, resulting in the delayed initiation of insulin therapy. We present a woman in her sixties with Hashimoto’s thyroiditis and a prior diagnosis of Sjögren syndrome who developed progressively worsening hyperglycemia and concurrent rheumatological symptoms, including chronic joint pain and stiffness, despite treatment with multiple noninsulin therapies. Her lean body habitus, absence of clinical insulin resistance, progressive glycemic deterioration, autoimmune background, and musculoskeletal manifestations prompted further evaluation, which revealed markedly elevated pancreatic autoantibodies, including glutamic acid decarboxylase (GAD-65), islet antigen-2 (IA-2), and zinc transporter 8 (ZnT8) antibodies, confirming the diagnosis of LADA with evolving β-cell failure. Transition to insulin-based therapy resulted in excellent glycemic control and was accompanied by substantial improvement in musculoskeletal symptoms, consistent with metabolic rather than inflammatory pathology. This case highlights the importance of recognizing LADA in patients with phenotypic discordance, autoimmune clustering, and systemic manifestations that improve with optimized glycemic control.

## Introduction

Latent autoimmune diabetes in adults (LADA) is a slowly progressive autoimmune form of diabetes characterized by adult onset, pancreatic autoantibodies, initial insulin independence followed by progressive β-cell failure and insulin dependence, and frequent coexistence with other autoimmune disorders [1-7]. It occupies a clinical and immunologic continuum between classic type 1 diabetes mellitus (T1DM) and type 2 diabetes mellitus (T2DM), often presenting with features that overlap both entities. As a result, LADA is frequently misclassified as T2DM at initial diagnosis, particularly in adults who do not present with acute insulin deficiency.

Epidemiologic studies suggest that LADA accounts for approximately 10% of cases initially diagnosed as T2DM, making it a relatively common but underrecognized form of autoimmune diabetes [2]. This diagnostic ambiguity is clinically important, as delayed recognition may lead to prolonged exposure to noninsulin antihyperglycemic therapies that are ineffective in the setting of evolving beta-cell failure. Such delays may accelerate loss of endogenous insulin secretion and contribute to suboptimal long-term metabolic outcomes.

Autoimmune clustering is a well-established feature of LADA and serves as an important clinical clue to diagnosis. Autoimmune thyroid disease, particularly Hashimoto thyroiditis, is among the most frequently associated comorbid conditions, and the presence of additional autoimmune disorders should raise suspicion for underlying autoimmune diabetes. Recognition of these associations is critical, as targeted pancreatic autoantibody testing can facilitate earlier diagnosis and appropriate classification.

Early identification of LADA enables the timely initiation of insulin therapy, which may reduce glucotoxicity, mitigate beta-cell stress, and help preserve residual insulin secretion. In addition to improving glycemic control, appropriate classification and management may reduce the risk of secondary complications, including musculoskeletal manifestations that have been increasingly recognized as part of the systemic burden of chronic hyperglycemia [8]. Collectively, these considerations underscore the importance of heightened clinical awareness of LADA and a low threshold for autoimmune evaluation in adults with atypical diabetes phenotypes.

## Case presentation

A woman in her sixties with a history of Hashimoto thyroiditis, Sjögren syndrome, and a three-year history of type 2 diabetes mellitus presented to the endocrinology clinic with progressively worsening glycemic control.

She had a body mass index of 20 kg/m² and lacked clinical features suggestive of insulin resistance. Since her initial diagnosis, she had been treated with metformin, pioglitazone, and dulaglutide; however, glycemic control continued to deteriorate despite adherence to therapy. At presentation, hemoglobin A1c was 9.0% (reference range 3.8-5.6%), indicating poor glycemic control. Given her lean phenotype, poor response to noninsulin antihyperglycemic agents, and underlying autoimmune history, LADA was strongly suspected. A comprehensive pancreatic autoantibody panel was ordered, insulin therapy was initiated, and all noninsulin antidiabetic agents were discontinued.

Laboratory evaluation at the time of presentation

Autoimmune testing demonstrated markedly elevated pancreatic autoantibodies (Table 1). The patient also had a pre-existing diagnosis of Hashimoto thyroiditis with thyroid peroxidase antibody positivity. The presence of multiple high-titer pancreatic autoantibodies in conjunction with low-normal endogenous insulin production supported the diagnosis of LADA.

**Table 1:** Autoimmune antibody profile (H) indicates a value above the reference range. Reference ranges are laboratory-specific. GAD-65 = glutamic acid decarboxylase; IA-2 = islet antigen-2; ZnT8 = zinc transporter 8

Test	Result	Reference range
Thyroid peroxidase antibody	154 IU/mL (H)	<35 IU/mL
Glutamic acid decarboxylase (GAD-65) antibody	>250 IU/mL (H)	<5 IU/mL
Islet antigen-2 (IA-2) antibody	>350 IU/mL (H)	<7.5 IU/mL
Zinc transporter 8 (ZnT8) antibody	175 IU/mL (H)	<15 IU/mL
Islet cell antibody (IgG)	Positive	Negative
C-peptide	1.15 ng/mL	1.10–5.50 ng/mL

Rheumatology evaluation

**Figure 1:**
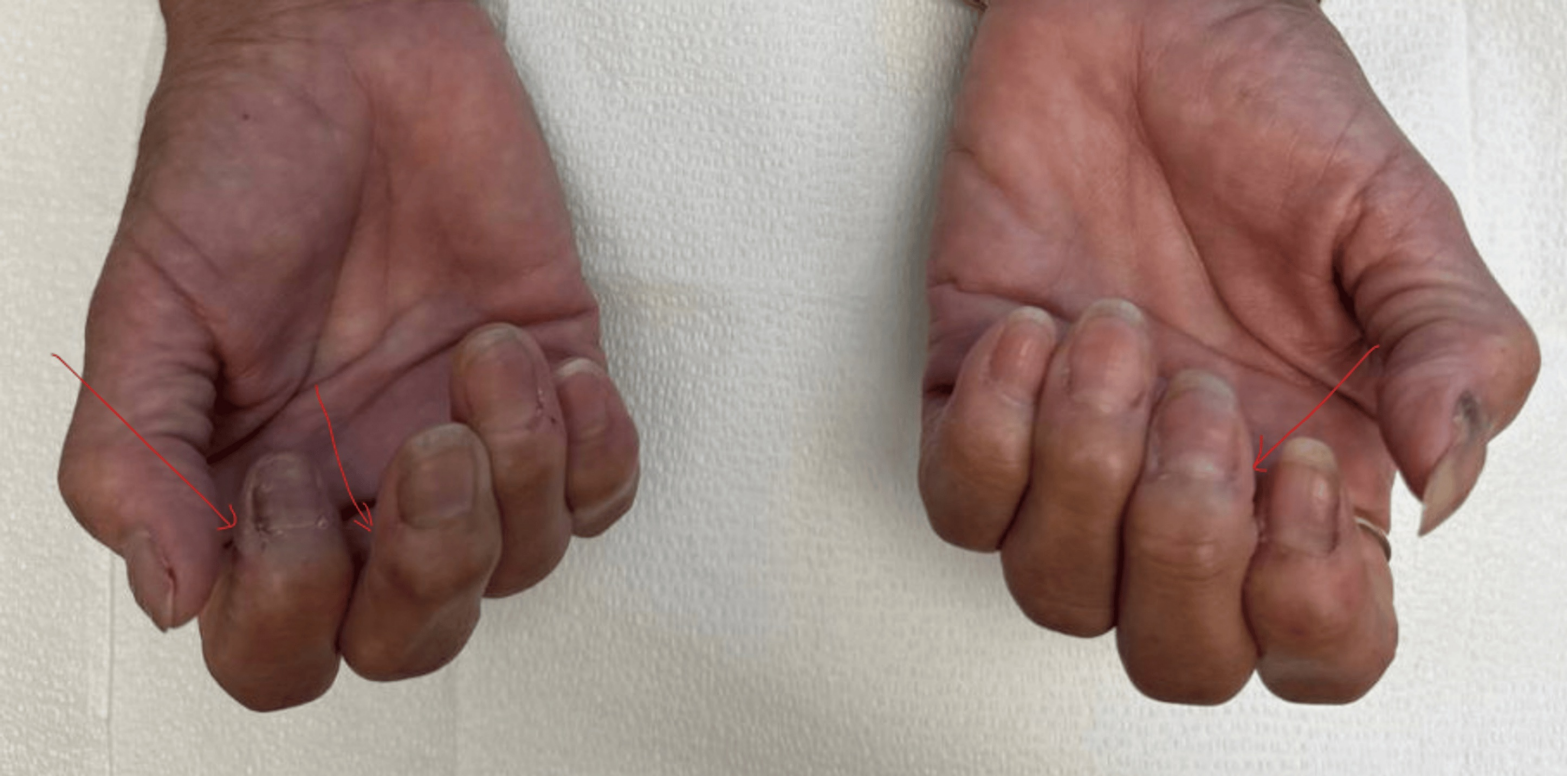
Fixed flexion deformity at the proximal interphalangeal joints resulting in limited finger mobility

Approximately two months after the initial endocrinology evaluation, the patient established care with rheumatology for chronic polyarthralgia, xerophthalmia, and xerostomia. Physical examination demonstrated multiple Heberden’s and Bouchard’s nodes, along with reduced range of motion of the fingers, raising concern for diabetic cheiroarthropathy (Figure 1). Further serologic evaluation demonstrated a negative antinuclear antibody (ANA), negative extractable nuclear antigen panel, and negative SSA (Ro) and SSB (La) antibodies, suggesting that her musculoskeletal symptoms were more likely attributable to metabolic complications of uncontrolled diabetes rather than active inflammatory rheumatologic disease.

Clinical course and outcomes

**Figure 2:**
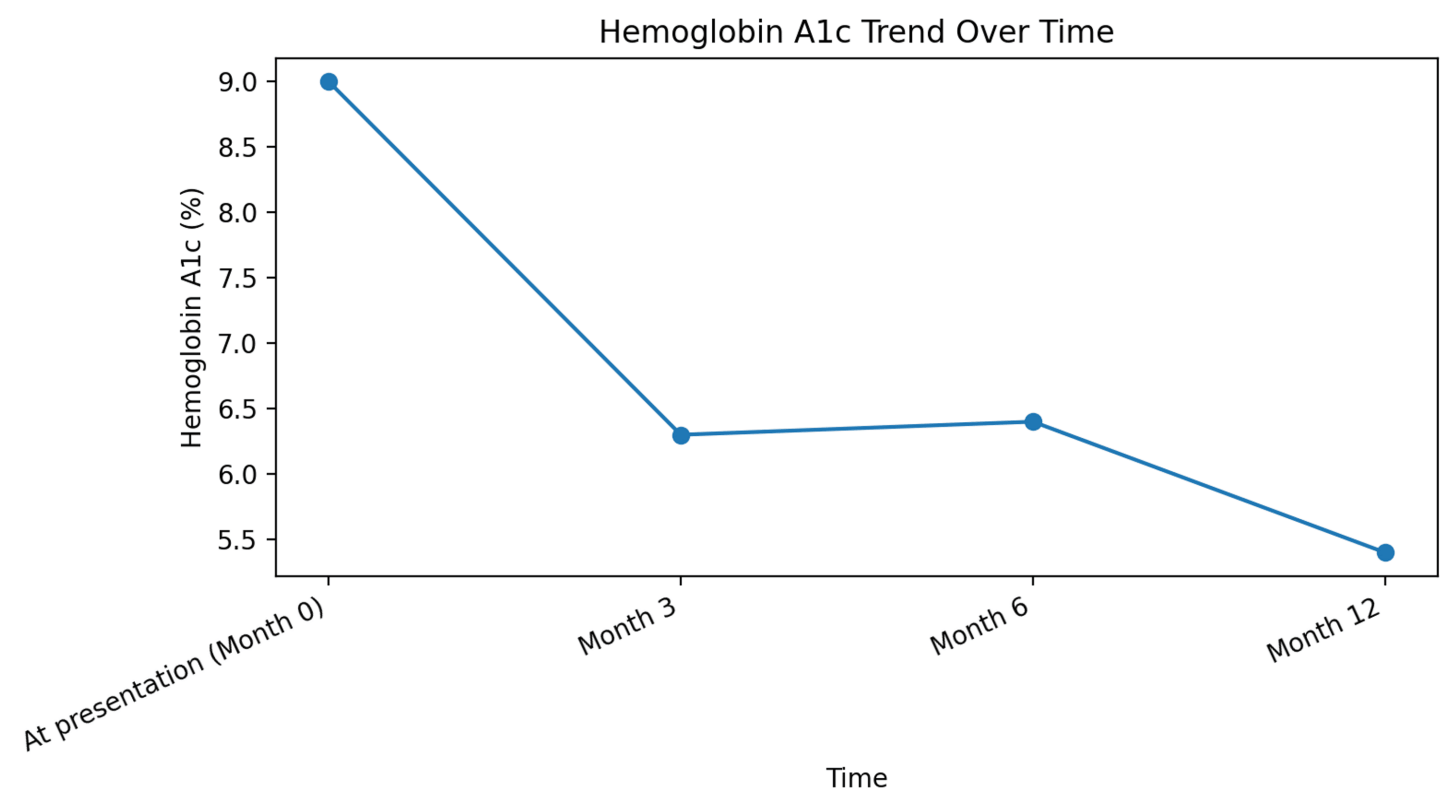
Trend in hemoglobin A1c over 12 months demonstrating progressive and sustained improvement in glycemic control following transition to insulin-based therapy Reference range for hemoglobin A1c: 3.8–5.6%

**Figure 3:**
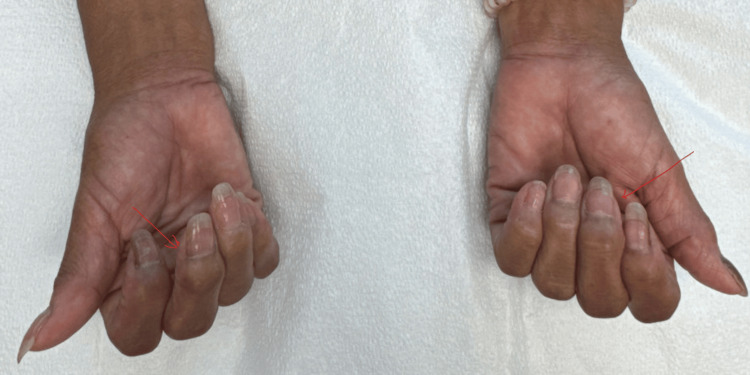
Improvement in fixed flexion deformity in the proximal interphalangeal joints

**Figure 4:**
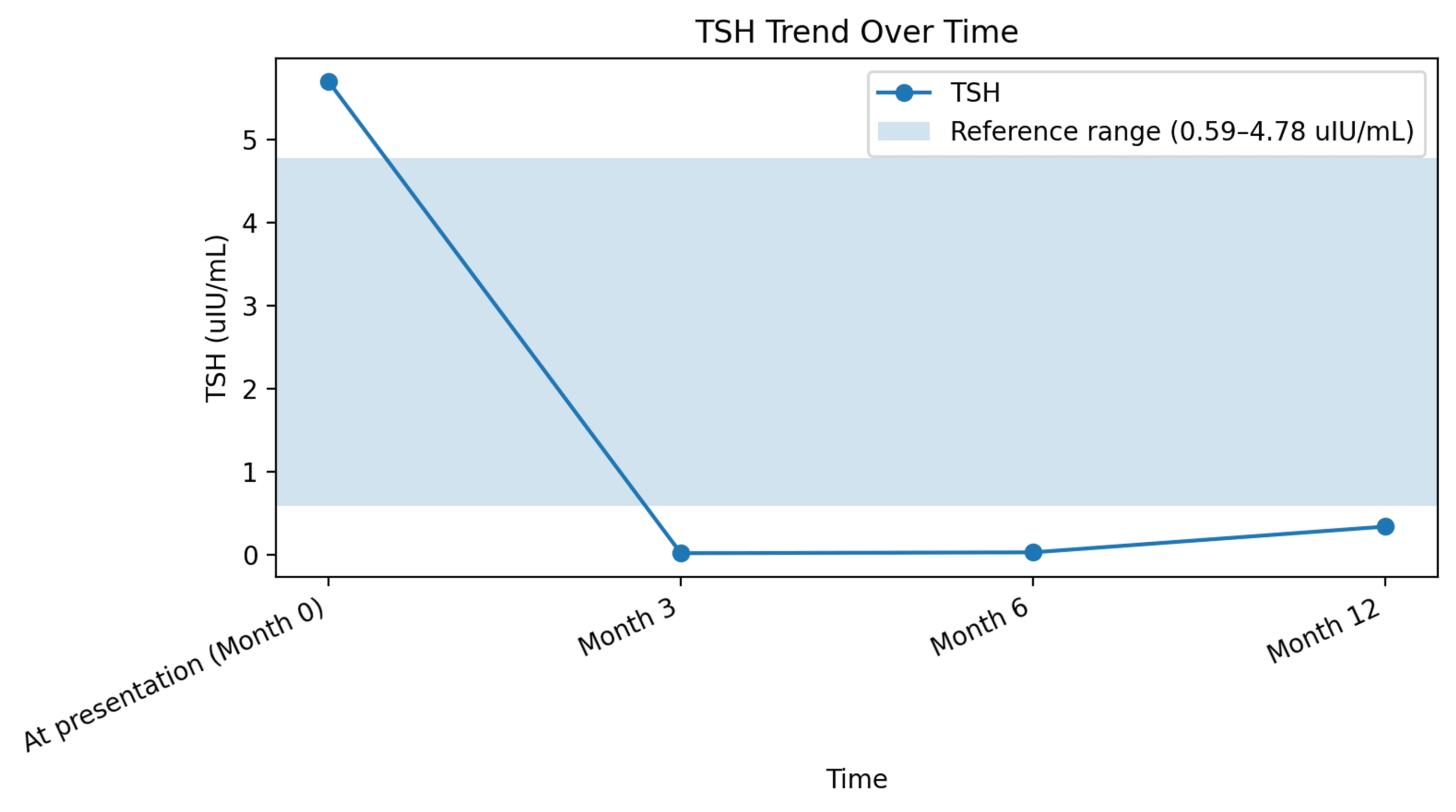
Trend in thyroid-stimulating hormone (TSH) levels over 12 months following the initiation and titration of levothyroxine therapy The shaded area represents the reference range (0.59–4.78 uIU/mL).

The patient transitioned to intensive insulin therapy and subsequently to insulin pump therapy, resulting in progressive improvement in glycemic control. Hemoglobin A1c improved from 9.0% at presentation to 5.4% at 12 months (reference range 3.8-5.6%) (Figure 2). Early initiation of insulin therapy reduced glucotoxicity and contributed to improved metabolic outcomes. Over approximately 12-14 months, the patient also reported significant improvement in joint pain and stiffness, correlating with improved metabolic control and consistent with prior observations in diabetic cheiroarthropathy (Figure 3). Thyroid function improved with levothyroxine therapy over a period of approximately 10-12 months, as reflected by improvement in thyroid-stimulating hormone (TSH) levels (Figure 4). At the time of manuscript preparation, the levothyroxine dose had been adjusted to achieve age-appropriate target TSH levels.

## Discussion

Latent autoimmune diabetes in adults represents a distinct form of autoimmune diabetes that occupies an intermediate position between classic type 1 diabetes mellitus and type 2 diabetes mellitus. Despite its autoimmune pathogenesis, LADA frequently presents in adulthood with a milder and more indolent course, leading to frequent misclassification as type 2 diabetes mellitus at initial diagnosis [1-3]. This diagnostic delay is clinically significant, as prolonged exposure to ineffective noninsulin therapies may accelerate beta-cell loss and worsen long-term metabolic outcomes.

In the present case, several features were discordant with a diagnosis of type 2 diabetes mellitus, including a lean body habitus, absence of clinical markers of insulin resistance, and progressive hyperglycemia despite adherence to multiple noninsulin antihyperglycemic agents. Importantly, the coexistence of autoimmune thyroid disease and Sjögren syndrome further increased the pretest probability of LADA, as autoimmune clustering is well recognized in this population. Autoimmune endocrinopathies, particularly Hashimoto thyroiditis, are among the most common comorbid conditions associated with LADA and should prompt consideration of pancreatic autoantibody testing in patients with atypical diabetes phenotypes.

The diagnosis of LADA was supported by the presence of multiple high-titer pancreatic autoantibodies, including GAD-65, IA-2, and ZnT8 antibodies [5,6]. Prior studies have demonstrated that both the number and titers of pancreatic autoantibodies correlate with the rate of beta-cell decline and earlier progression to insulin dependence. Patients with multiple autoantibodies, as seen in this case, are therefore more likely to experience rapid deterioration of endogenous insulin secretion, underscoring the importance of early and accurate classification.

Early initiation of insulin therapy remains a cornerstone of LADA management. Insulin therapy reduces glucotoxicity, mitigates ongoing beta-cell stress, and may help preserve residual endogenous insulin production [7]. In contrast, prolonged reliance on noninsulin therapies alone may delay appropriate treatment and contribute to progressive metabolic decompensation. In this patient, transition to insulin-based therapy, including subsequent insulin pump use, resulted in marked and sustained improvement in glycemic control, supporting the benefit of timely insulin initiation in LADA.

Beyond glycemic outcomes, this case also highlights the broader systemic consequences of prolonged hyperglycemia in autoimmune diabetes. The patient’s musculoskeletal manifestations, initially concerning for inflammatory rheumatologic disease, were ultimately more consistent with diabetic cheiroarthropathy [8]. This condition is a recognized complication of chronic hyperglycemia and is thought to result from nonenzymatic glycation of connective tissue proteins, leading to joint stiffness and reduced mobility [8]. Improvement in joint symptoms following the optimization of glycemic control further supports the metabolic rather than inflammatory etiology of her musculoskeletal findings and underscores the multisystem benefits of effective diabetes management [8].

This case reinforces the need for heightened clinical awareness of LADA, particularly in patients with autoimmune comorbidities and atypical metabolic features. Early recognition and appropriate therapy not only improve glycemic outcomes but may also prevent or reverse secondary complications that significantly impact quality of life.

## Conclusions

Latent autoimmune diabetes in adults (LADA) should be strongly considered in patients with presumed type 2 diabetes mellitus who exhibit phenotypic discordance, autoimmune clustering, and poor response to noninsulin therapies. Early pancreatic autoantibody testing facilitates timely diagnosis and prevents delays in appropriate treatment.

Prompt initiation of insulin therapy is essential to reduce glucotoxicity, preserve residual beta-cell function, and prevent long-term metabolic and musculoskeletal complications associated with LADA. Increased clinical vigilance and early intervention can substantially improve patient outcomes.
